# A randomized, double-blind, placebo-controlled phase III study evaluating the preventive effect of diclofenac cream on capecitabine-related hand-foot syndrome: study protocol of J-SUPPORT2401/JORTC-SUP06 (J-DIRECT)

**DOI:** 10.1007/s10147-025-02789-z

**Published:** 2025-05-14

**Authors:** Yohei Iimura, Hiroshi Ishiguro, Hironobu Hashimoto, Masanori Nojima, Shunsuke Oyamada, Keita Mori, Keisuke Ariyoshi, Seiichiro Kuroda, Satoshi Hirakawa, Noriko Fujiwara, Tomoya Yokota, Sadamoto Zenda, Hiromichi Matsuoka, Narikazu Boku

**Affiliations:** 1 Department of Pharmacy, The IMSUT Hospital, The Institute of Medical Science, The University of Tokyo, 4-6-1, Shirokanedai, Minato-ku, Tokyo 108-8639 Japan; 2 Breast Oncology Service, Saitama Medical University International Medical Center, Saitama, Japan; 3 Department of Pharmacy, National Cancer Center Hospital, Tokyo, Japan; 4 Center for Translational Research, The Institute of Medical Science, The University of Tokyo, Tokyo, Japan; 5 Japanese Organisation for Research and Treatment of Cancer Data Centre, NPO, Tokyo, Japan; 6 Clinical Research Center, Shizuoka Cancer Center, Shizuoka, Japan; 7 Department of Care in Cancer, Seirei Hamamatsu General Hospital, Shizuoka, Japan; 8 Department of Palliative Medicine and Advanced Clinical Oncology, IMSUT Hospital of the Institute of Medical Science, The University of Tokyo, Tokyo, Japan; 9 Division of Gastrointestinal Oncology, Shizuoka Cancer Center, Shizuoka, Japan; 10 Innovation Center for Supportive, Palliative and Psychosocial Care, National Cancer Center Hospital, Tokyo, Japan; 11 Department of Psycho-Oncology, National Cancer Center Hospital, National Cancer Center Japan, Tokyo, Japan; 12 Department of Oncology and General Medicine, The Institute of Medical Science Hospital, The University of Tokyo, Tokyo, Japan

**Keywords:** Capecitabine-induced hand-foot syndrome, Topical diclofenac cream, Protocol

## Abstract

**Background:**

Clinical evidence on preventive therapy for capecitabine-induced hand-foot syndrome (HFS) is limited, and moisturizing and avoiding local pressure are recommended in guidelines. Although the precise pathogenesis and mechanisms of HFS remain unclear, inflammatory reactions are thought to be involved. The preventive effects of topical diclofenac gel have been reported from India. However, the trial did not evaluate its preventive effect for the sole, and the HFS incidence in the control group was lower than that in previous reports. Therefore, this study aims to confirm the preventive effects of diclofenac sodium 0.1% cream for capecitabine-induced HFS.

**Methods:**

This is a multicenter, randomized, double-blind, placebo-controlled, phase 3 trial. Patients scheduled to receive capecitabine-containing chemotherapy are enrolled, and participants are prophylactically treated with topical diclofenac sodium 0.1% cream or placebo alongside standard preventive therapy. The primary endpoint is an incidence of grade 2 HFS within 3 months. The secondary endpoints include time to onset of HFS, incidences of dose reduction, schedule delay, discontinuation caused by capecitabine-induced HFS, dose intensity of capecitabine, an incidence of grade ≥ 2 peripheral sensory neuropathy, incidences of other capecitabine-related adverse events (nausea, vomiting, appetite loss, diarrhea, oral mucositis, pigmentation, abnormality of liver and renal functions, and neutropenia).

**Discussion:**

If this study meets the primary endpoint, a new standard preventive therapy for HFS will be established. Moreover, the use of topical diclofenac cream alongside high-dose capecitabine may enhance chemotherapy efficacy.

## Introduction

Hand-foot syndrome (HFS) is the most common adverse event induced by capecitabine, often deteriorating patient’s quality of life (QOL) [1]. In randomized-controlled trials (RCTs) of systemic chemotherapy for colorectal cancer, the incidence of any grade HFS caused by capecitabine have been reported to be approximately 30% to 80% [2–10], whereas real-world data suggest that it is over 90% [11]. Severe HFS causes symptoms, such as swelling, blisters, desquamation, and ulcers, often leading to treatment interruptions, delays in the treatment schedule, dose reduction, and discontinuation of capecitabine. One study showed that 17–24% of patients with metastatic colorectal cancer receiving capecitabine monotherapy required dose and schedule modifications due to HFS [12]. Additionally, while it has been reported that dose intensity of capecitabine influences the outcomes of adjuvant chemotherapy for colon cancer [13], moderate or severe HFS requires treatment modifications (interruption and/or dose reduction) [14]. Therefore, managing HFS is crucial not only for maintaining patient’s QOL but also for ensuring the therapeutic efficacy of capecitabine [15]. However, a standard preventive therapy has not been established due to inconsistent results regarding the effectiveness of topical urea [16, 17], exfoliating agents [18], celecoxib [19, 20], vitamin E [21], and pyridoxine [22] for HFS. Currently, moisturizing and avoiding local pressure [23–25] are recommended as standard management methods for preventing capecitabine-induced HFS [26].

The mechanism of HFS involves the inhibition of the skin basal cell proliferation, drug secretion from the eccrine sweat glands, the involvement of drug degradation products [15, 27], and an inflammatory response triggered by interleukin (IL)-1a, IL-1b, IL-6, and reactive oxygen species [28]. Based on this rationale, a large phase III study demonstrated that celecoxib has a preventive effect on HFS. However, its clinical use has been limited because of concerns about adverse events caused by the long-term use of non-steroidal anti-inflammatory drugs (NSAIDs). Moreover, clinical trials have evaluated the preventive effects of medium-class topical steroids [29] and topical diclofenac [30]. Corticosteroids exert their anti-inflammatory effects by inhibiting the release of chemical mediators. Oral dexamethasone (8 mg/day, followed by tapering) was administered to patients with pegylated liposomal doxorubicin-induced HFS in a prospective study [31], which reported that patients receiving dexamethasone required fewer treatment modifications, such as schedule delays or dose reductions than those not receiving dexamethasone. Other case reports have suggested that corticosteroids may be effective against cytarabine- and vinorelbine-induced HFS [32, 33]. NSAIDs inhibit the production of cyclooxygenase (COX)-2, which is believed to contribute to the development of HFS [34]. Thus, anti-inflammatory agents may help prevent HFS caused by cytotoxic anti-tumor agents. Although the effectiveness of oral COX-2 inhibitors has been reported [19, 20], safety concerns remain.

Since topical formulations offer better safety by reducing the systemic effects of NSAIDs, the topical use of anti-inflammatory agents, such as corticosteroids and NSAIDs, is expected to prevent HFS without major safety concerns. Recently, the preventive efficacy of topical diclofenac was reported in India (D-TORCH study) [35], demonstrating that the prophylactic administration of topical diclofenac reduced the frequency of grade ≥ 2 HFS by 11.2% (3.8% vs 15.0%). However, the incidence of HFS in the placebo group of the D-TORCH study was substantially lower than that in other Asian studies (Table 1). Additionally, the D-TORCH study evaluated the effectiveness of topical diclofenac only on the hands, not the soles. Furthermore, while the study used a diclofenac gel base, the isopropanol in the gel may cause skin dryness and irritation, potentially worsening HFS symptoms. A cream-based formulation is generally considered more suitable than a gel for preventing HFS.

**Table 1:** Difference of the incidence of HFS induced by capecitabine in India (D-TORCH study) and other Asian countries in randomized-controlled trials

					Incidence rate of HFS (any grade)		Incidence rate of HFS (grade 2 or 3)		Incidence rate of HFS (≥ grade 3)	
			Number of patients		%		%		%	
Country	Intervention	Blind	Intervention	Control	Intervention	Control	Intervention	Control	Intervention	Control
China 2010 [19]	Celecoxib	No	51	50	29	52	11.76	30	1.96	10
China 2012 [20]	Celecoxib	No	68	71	57.4	74.6	14.7	29.6	2.9	8.5
South Korea 2010 [44]	Pyridoxine	Yes	180	180	64	76.1	12	15.4	3.3	5
Japan 2014 [45]	Pyridoxine	No	30	30	NA	NA	60	60	NA	NA
Singapore 2017 [46]	Pyridoxine	Yes	105	105	61	66	32	37	4	2
Japan 2018 [47]	Pyridoxine	No	66	67	77	69	34	34	4	4
Japan 2020 [48]	Eppikajutsuto (Kampo medicine)	No	10	12	70	83.3	40	50	20	8.3
China 2021 [18]	Lithium containing topical and moisturizing cream	Yes	51	54	56.8	75.9	4.8	16.7	6.0	16.7
India 2020 [36]	Structured teaching module	Yes	135	134	64.4	62.5	33.3	32.8	5.2	6.8
India 2024 [35]	Topical diclofenac	Yes	130	133	6.1	18.1	3.8	15	2.3	5.3

This multicenter, randomized, double-blind, placebo-controlled, phase 3 trial aims to confirm the preventive effect of diclofenac sodium 0.1% cream for capecitabine-induced HFS.

## Methods

### Study design

This is a multicenter, randomized, double-blind, placebo-controlled, phase 3 study. The study protocol was reviewed and approved by the certified Clinical Research Review Board of the University of Tokyo (approval number 2024505SP). The study is being conducted in compliance with the Clinical Trials Act in Japan and registered in the Japan Registry of Clinical Trials (jRCT) as jRCTs031240705.

### Participants

The following patients are included in this study: (i) patients with colorectal, breast, or gastric cancer planned to receive chemotherapy containing capecitabine (≥ 800 mg/m^2^ b.i.d); (ii) Eastern Cooperative Oncology Group performance status of 0–1, (iii) aged ≥ 18 years; and (iv) providing written informed consent. Exclusion criteria are as follows: (i) history of aspirin-induced asthma; (ii) presence of other skin diseases, skin lesions due to chemotherapy on the palms or soles, or dermatitis that makes it difficult to evaluate the efficacy of the study drug; (iii) history of hypersensitivity to local diclofenac; (iv) conditions requiring the constant use of steroids or NSAIDs; (v) scheduled to receive other anti-tumor agents (e.g., multi-kinase inhibitors for anti-angiogenesis) that may interfere with the evaluation of efficacy of the study drug; (vii) pregnancy, breast feeding, or inability to adhere to contraception during the study period; and (viii) sensory palsy in the palms or soles.

### Randomization

After confirming eligibility, patients are registered using an independent central registration system. They are randomized in a 1:1 ratio to receive either diclofenac sodium 0.1% cream or a placebo cream using the minimization method, adjusted for sex (male vs. female), planned dose of capecitabine (≥ 800 mg/m^2^, < 1000 mg/m^2^, ≥ 1000, < 1250 mg/m^2^, ≥ 1250 mg/m^2^), and participating institution.

### Chemotherapy

Combination with other anti-tumor agents (e.g. oxaliplatin) and radiation (except for the palms and soles) is allowed if it includes capecitabine (≥ 800 mg/m^2^). However, multi-kinase inhibitors (e.g., lapatinib) will not be allowed. Dose interruptions, delays, reductions, and discontinuations are allowed at each physician’s discretion.

### Intervention

Both diclofenac sodium 0.1% cream and placebo are white cream products. The hydrophilic cream, which is the base material of diclofenac sodium 0.1% cream, is selected as the placebo. Since there is no noticeable difference in appearance or odor, double-blindness is ensured. Topical diclofenac sodium 0.1% cream or placebo is applied to the palms and soles twice daily in the morning and evening. More than one fingertip unit (one fingertip unit provides 0.5 g) of the study drug is applied to each palm and sole. This preventive treatment is initiated in the evening on day 1 and continued throughout the study period. All patients receive standard self-care education (e.g., maintaining cleanliness, avoiding strenuous exercise and pressure, and using sun protection) at the start of chemotherapy. Clinical pharmacists monitor the volume of study drugs used by checking the remaining amount and educate the patients to improve their adherence, if necessary.

### Prohibited therapy and drugs

The following treatments or concomitant use of drugs are prohibited: (i) cancer treatment other than the capecitabine-based chemotherapy (localized radiation therapy excluding the palm or sole is allowed), (ii) continuous use of other topical corticosteroids or NSAIDs on the palms or soles, (iii) continuous use of NSAIDs, (iv) continuous use of pyridoxine, and (v) use of multi-kinase inhibitors.

### Discontinuation of interventions

Interventions are discontinued if any of the following events occurs: (i) discontinuation of capecitabine for any reason, (ii) withdrawal of consent, (iii) serious adverse events induced by the study drugs, and (iv) use of prohibited therapies or drugs. Optimal medical care without the study drugs is guaranteed to participants who discontinue or deviate from the intervention protocols.

### Evaluation

Patients complete a self-report check sheet daily to document symptoms of HFS and adherence to the study drug. Patient’s symptoms and laboratory tests results are reviewed at every visit, and the severity of HFS is assessed by the clinical pharmacist and attending physician by referring to the self-reported adverse events (Tables 2 and 3). Evaluators will be undergo regular training to ensure data quality.

**Table 2:** Assessment schedule

			Chemotherapy period (weeks)			
		Pretreatment	3	6	9	12
Performance status		✓	✓	✓	✓	✓
Blood test		✓	✓	✓	✓	✓
Hand-foot syndrome			✓	✓	✓	✓
Peripheral sensory neuropathy			✓	✓	✓	✓
Nausea			✓	✓	✓	✓
Vomiting			✓	✓	✓	✓
Appetite loss			✓	✓	✓	✓
Diarrhea			✓	✓	✓	✓
Oral mucositis			✓	✓	✓	✓
Pigmentation			✓	✓	✓	✓
Abnormality of liver function			✓	✓	✓	✓
Abnormality of renal function			✓	✓	✓	✓
Neutropenia			✓	✓	✓	✓
Self-report (adverse events)			✓	✓	✓	✓
Self-report (self-adherence)			✓	✓	✓	✓
Dose of chemotherapy			✓	✓	✓	✓
Postponement of chemotherapy			✓	✓	✓	✓
Discontinuation of chemotherapy			✓	✓	✓	✓

**Table 3:**
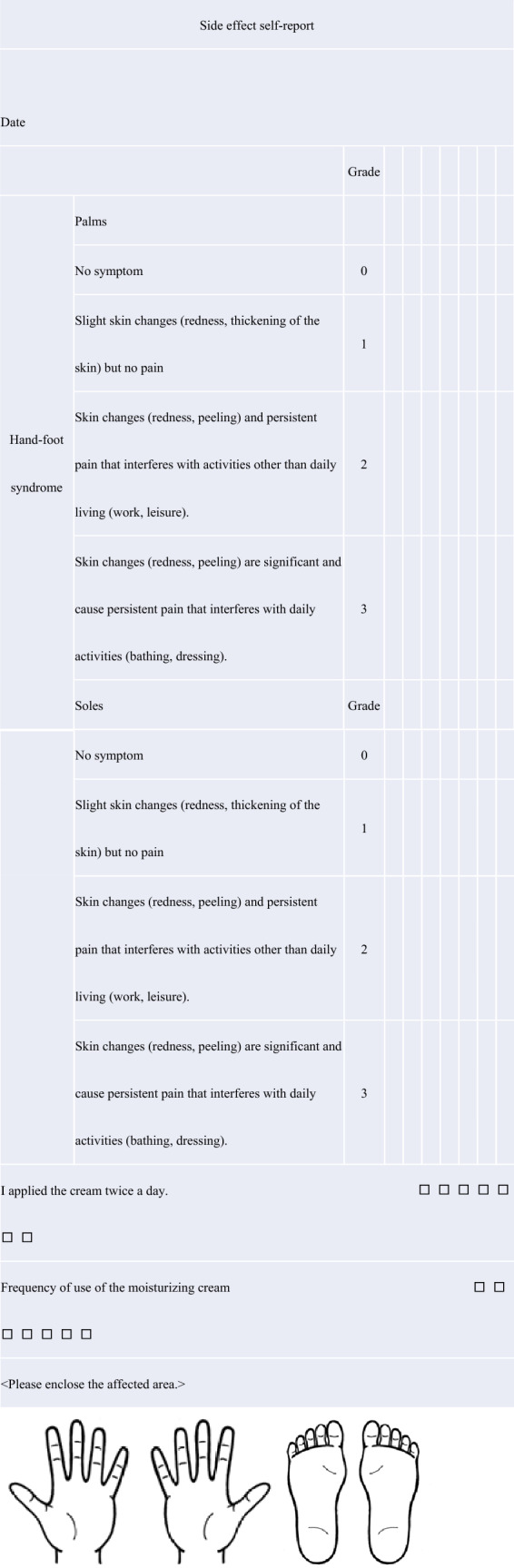
Self-report of hand-foot syndrome and self-adherence check sheet

### Endpoints

The primary endpoint is the incidence of grade ≥ 2 HFS within 3 months. Secondary endpoints include time to onset of grade ≥ 2 HFS, time to onset of grade ≥ 1 HFS within 3 months, incidences of dose reduction, schedule delay, discontinuation due to capecitabine-induced HFS, an incidence of grade ≥ 2 peripheral sensory neuropathy (subgroup analysis will be performed in patients treated with oxaliplatin), and incidences of any grade capecitabine-related adverse events (nausea, vomiting, appetite loss, diarrhea, oral mucositis, pigmentation, abnormality of liver and renal functions, and neutropenia).

### Sample size calculation

Based on previous RCTs evaluating the preventive effect for HFS, the incidence of grade ≥ 2 HFS within 3 months in the control group without preventive measures was approximately 40% [19, 20, 36]. Accordingly, the incidence of grade ≥ 2 HFS in the placebo arm is assumed to be 40%. It is expected that diclofenac sodium 0.1% cream will suppress grade ≥ 2 HFS within 3 months by 15%, resulting in an anticipated incidence rate of 25%. With a power of 80% and one-sided α of 5%, the required sample size is 160 patients in each arm. Therefore, the target number of enrolled patients is 320. No interim analysis is planned. Sample size calculations will be performed using SAS Studio 3.8.

### Management of severe HFS occurrence

In the event of a severe HFS, the study drug application will be discontinued immediately, and treatment with highly potent topical steroid-based therapy should be prioritized in both groups. The principal investigator and research assistants will administer appropriate treatment to the study participants, take the best possible measures, and document the adverse event in the medical record or case report form. Observation of the study participants after adverse events, including illnesses, will be followed up until the event disappears or until the principal investigator or research assistant determines that follow-up is no longer necessary.

## Discussion

This study investigates the preventive effect of topical diclofenac sodium 0.1% cream for capecitabine-induced HFS.

According to the Common Terminology Criteria for Adverse Events version 5.0, grade 2 HFS is defined as a painful skin change, including delamination, blistering, and bleeding, which significantly reduces patients’ QOL. It often leads to treatment interruption, dose reduction, or even discontinuation of capecitabine. Preventing grade ≥ 2 HFS is expected not only improve patients’ QOL but also maintain treatment intensity. As reducing the overall incidence of HFS is more clinically meaningful than merely delaying its onset, the incidence of grade ≥ 2 HFS within 3 months is set as the primary endpoint. The evaluation period is set at 3 months, because, in patients receiving eight courses (6 months) of capecitabine plus oxaliplatin as adjuvant chemotherapy after surgery of colorectal cancer, the incidence of HFS increased until the 4th course (at 3 months after the start of treatment) and then plateaued [37]. In addition, the median time of onset of grade ≥ 2 HFS induced by fluoropyrimidine anticancer drugs has been reported to be approximately 100 days [38, 39]. Therefore, evaluating the incidence of grade ≥ 2 HFS at 3 months is deemed appropriate as the primary endpoint. The majority of participants are expected to be patients with colorectal cancer, receiving capecitabine as adjuvant chemotherapy, which is typically recommended for at least 4 courses (3 months). In Japan, capecitabine plus oxaliplatin in combination with bevacizumab is the most commonly used palliative chemotherapy, with a reported median progression-free survival time of approximately 10 months [39]. The second major population will consist of patients with breast cancer. The median progression-free survival of capecitabine monotherapy in patients with advanced breast cancer has been reported as approximately 3.2 months [7]. Thus, it is expected that most participants will continue capecitabine for 3 months, facilitating the evaluation of the primary endpoint.

The expectation of a 15% reduction in the frequency of HFS is based on a study using celecoxib as the study drug, where the incidence of grade ≥ 2 HFS was reduced by approximately 20% compared with the placebo group [19, 20]. Based on these reports, we expect that a 15% reduction in incidence of HFS would provide a lower risk and equally effective preventive method compared to celecoxib.

The secondary endpoints include the time to onset of grade ≥ 2 HFS, the time to onset of grade ≥ 1 HFS within 3 months, incidences of dose reduction, schedule delay, discontinuation due to capecitabine-induced HFS, an incidence of grade ≥ 2 peripheral sensory neuropathy, and an incidence of any grade capecitabine-related adverse events (nausea, vomiting, appetite loss, diarrhea, oral mucositis, pigmentation, abnormality of liver and renal functions, and neutropenia). It is anticipated that preventing HFS with diclofenac cream may improve the dose intensity of capecitabine. However, there is a concern that capecitabine-related adverse events (nausea, vomiting, anorexia, diarrhea, mucositis, pigmentation, liver dysfunction, renal dysfunction, myelosuppression) may be exacerbated. Peripheral sensory neuropathy is also included as a secondary endpoint, assuming that absence of grade ≥ 2 HFS may alleviate some symptoms of neuropathy induced by oxaliplatin. This study will also include the following subgroup analyses: (i) frequency of grade ≥ 2 peripheral sensory neuropathy in patients treated with oxaliplatin, (ii) cumulative dose of capecitabine in the diclofenac and placebo groups, and (iii) frequency and severity of HFS, assessed separately for the palms and soles.

**Fig. 1:**
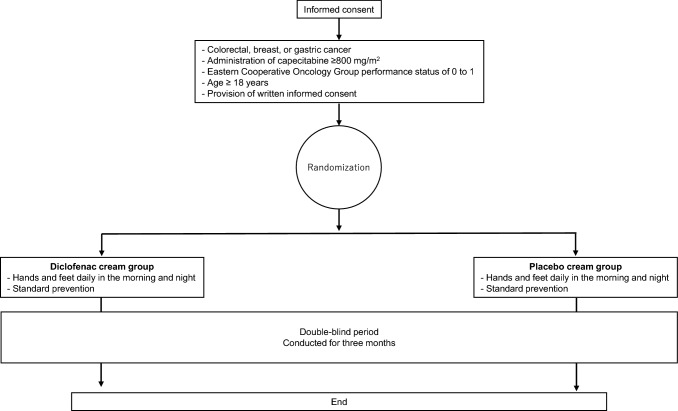
Study scheme. Randomization will be conducted using a minimization method, adjusting for sex (male vs. female) and planned dose of capecitabine ([1] ≥ 800 mg/m^2^, < 1000 mg/m^2^, [2] ≥ 1000, < 1250 mg/m^2^, [3] ≥ 1250 mg/m^2^). Topical hydrocortisone butyrate 0.1%, diclofenac sodium 0.1% cream, or placebo cream will be applied to the palms and soles daily in the morning and evening for 3 months. To maintain self-adherence, all patients will receive standard self-care education at the start of chemotherapy, and the number of study agents used regularly by clinical pharmacists will be confirmed. Clinical pharmacists will regularly educate patients to improve their adherence to intervention protocols

The stratification factors for randomization include the dose of capecitabine and sex, as the incidence of HFS depends on capecitabine dose. Additionally, capecitabine-related adverse events differ between sexes [40, 41]. Body composition also varies between women and men [42, 43]. A previous study showed that female patients with colorectal cancer experienced more dose-limiting toxicity than male patients when capecitabine was administered based on body surface area [41] (Fig. 1).

If the J-DIRECT study demonstrates the prophylactic efficacy of diclofenac sodium 0.1% cream against capecitabine-induced HFS, it could establish a new preventive therapy for HFS. In addition, the suppression of HFS with topical diclofenac cream may improve the efficacy of high-dose intensity capecitabine-based chemotherapy.

## Data Availability

The data that support the findings of this study are available from the corresponding author, YI, upon reasonable request. Only clinical data managers at the central data center have access to reported case data through the EDC system during the study period. The data manager will transfer the final data set to the principal investigator and the data will be stored in the electronic format.

## Abbreviations

CAPEOX: Capecitabine plus intravenous oxaliplatin
FOLFOX: 5-Fluorouracil and leucovorin plus oxaliplatin
HFS: Hand-foot syndrome
jRCT: Japan Registry of Clinical Trials
QOL: Quality of life
RCT: Randomized-controlled trial

## Declarations

### Confidentiality

Personal information, such as name, address, and medical ID, will be not collected.

### Trial status

The study will be initiated in April 2025. We expect to complete the recruitment by March 2027.
